# One-stage laparoscopic resection for a large gastric gastrointestinal stromal tumor and synchronous liver metastases following preoperative imatinib therapy: A case report

**DOI:** 10.3892/ol.2013.1197

**Published:** 2013-02-18

**Authors:** FENG CAO, JIA LI, ANG LI, YU FANG, FEI LI

**Affiliations:** Department of General Surgery, Xuanwu Hospital, Capital Medical University, Beijing 100053, P.R. China

**Keywords:** gastrointestinal stromal tumor, laparoscopic, imatinib, preoperative therapy

## Abstract

Laparoscopic partial gastrectomy without lymph node dissection has been accepted worldwide for the treatment of small gastric gastrointestinal stromal tumors (GISTs). However, the role of laparoscopic surgery in the treatment of large gastric GISTs remains under debate due to the risk of tumor spillage or rupture of the tumor capsule leading to peritoneal seeding. To the best of our knowledge, one-stage laparoscopic resection for a large gastric GIST and synchronous liver metastases following preoperative imatinib therapy has not been previously reported. Here, we present our initial experience of this method of treatment.

## Introduction

Gastric gastrointestinal stromal tumor (GIST) may be cured by sleeve resection without lymph node dissection. Laparoscopic surgery for GIST has been established as a less invasive option, provided that it is performed by experienced laparoscopic surgeons (1–4). However, laparoscopic resection for large gastric GISTs remains under debate due to the high risk of intraoperative tumor rupture. Liver metastases are common in advanced or unresectable GISTs. Patients with multiple liver metastases carry the risk of severe postoperative functional deficit if massive hepatectomy is performed. In the present study we present a case of large gastric GIST and synchronous liver metastases in a patient receiving one-stage laparoscopic resection following preoperative imatinib treatment. The study was approved by the Ethics Committee of Xuanwu Hospital, Capital Medical University, Beijing, China. Written informed consent was obtained from the patient.

## Case report

**Figure 1:**
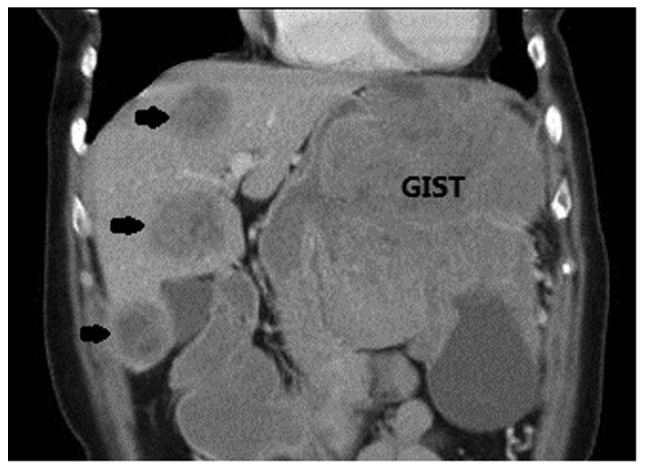
Enhanced CT scan (coronal reconstruction) showed a giant mass in the stomach and three low-density regions in different liver segments (arrow) with evident heterogeneous enhancement. CT, computed tomography; GIST, gastrointestinal stromal tumor.

A 63-year-old female was admitted to the Department of General Surgery, Xuanwu Hospital, Beijing, China, complaining of melena for 5 days. Following resuscitation, gastric endoscopy was performed and a submucosal lesion in the body of the stomach was reported. A biopsy confirmed the diagnosis of gastric GIST with c-kit exon 11 mutation. Computed tomography (CT) of the abdomen showed an intramural GIST ∼15×10 cm in size with multiple liver metastases (Fig. 1). The CT value was 22 and 46 HU prior to and following contrast introduction, respectively. Tumor markers were within the normal range.

**Figure 2:**
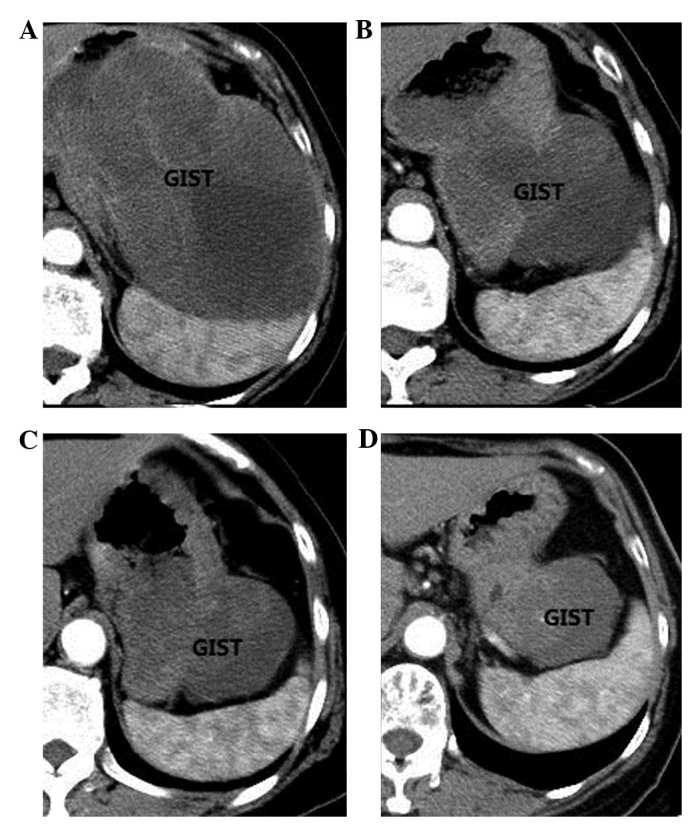
Enhanced CT scan of GIST (A) before imatinib treatment, and (B) 2, (C) 4 and (D) 6 months after therapy revealed a significant decrease in the tumor size. CT, computed tomography; GIST, gastrointestinal stromal tumor.

**Figure 3:**
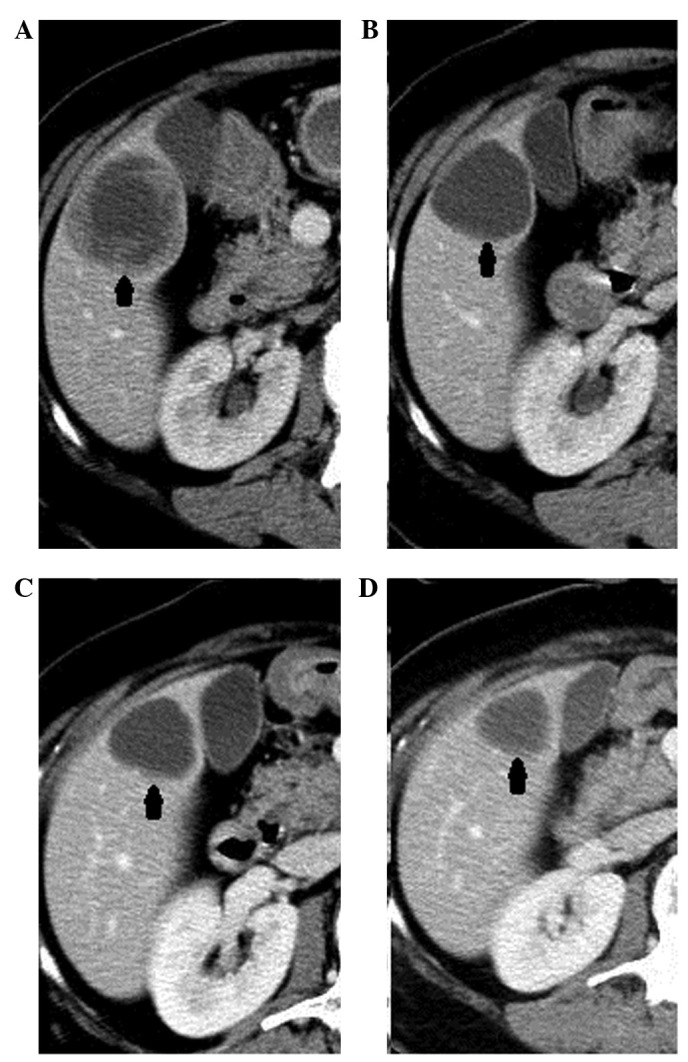
Enhanced CT scan of liver metastasis (arrow) before imatinib treatment (A), and (B) 2, (C) 4 and (D) 6 months after therapy revealed a significant decrease in the tumor size and CT value. CT, computed tomography.

Preoperative imatinib (400 mg/day) therapy was administered to this patient. The clinical and morphologic response was monitored by physical examination and CT scan every two months (Figs. 2 and 3). The size of the tumor decreased markedly and the CT value declined. No symptoms of upper gastrointestinal bleeding recurred during the treatment.

**Figure 4:**
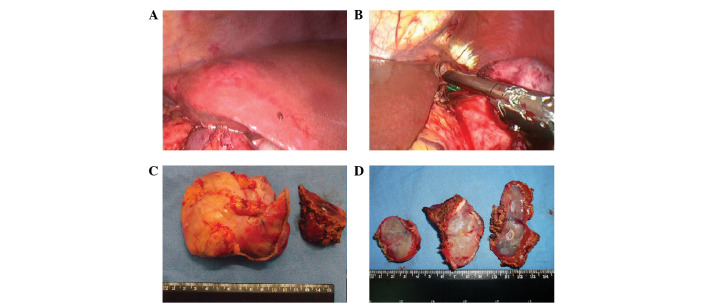
One-stage laparoscopic resection for (A) liver metastases and (B) gastric gastrointestinal stromal tumor (GIST). Resected specimens from (C) stomach and (D) liver.

After 6 months’ preoperative treatment, a partial response was achieved according to Choi criteria (5). Laparoscopic surgery was subsequently performed to remove the gastric GIST and liver metastases synchronously (Fig. 4). Laparoscopic sleeve resection was performed to resect the gastric tumor using two Endo GIA staplers (Ethicon, Johnson and Johnson, Cincinnati, OH, USA). In order to not disrupt the serosal surface and cause tumor spillage, the mass was grasped and gently elevated with an atraumatic bowel grasper during the operation. Liver metastases enucleation was then performed using the LigaSure system (Valleylab, Boulder, CO, USA). All the specimens were retrieved in an endobag without spillage through a muscle-splitting incision in the left flank. The surgery time was 185 min and the estimated blood loss was ∼150 ml. The recovery of the patient was uneventful.

**Figure 5:**
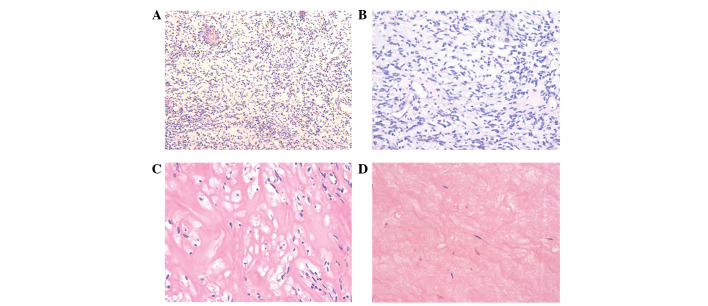
Pathological examination of gastric gastrointestinal stromal tumor (GIST); magnification, (A) ×200; (B) ×400, pre-treatment; (C) ×400, post-treatment. Pathological examination of liver metastasis (D); magnification, ×400, post-treatment. H&E staining.

Pathological evaluation of the stomach specimen revealed that the structure of the mucosa was normal, and that the tumor mass was present in the muscular layer. Microscopically, although tumor cells with sharp spindles were detected in certain areas, the number had declined significantly compared with that in the biopsy specimens. Many large areas of hyaline degeneration were found with mitotic figures <5 per 50 high-power fields (Fig. 5). The tumor margins were negative in both the stomach and liver specimens. The immunohistochemistry study showed that cells were positive for CD117 and CD34, and negative for SMA, desmin and s-100.

Four weeks following the surgery, imatimib treatment was resumed at 400 mg/day. Eleven months following the surgical resection, the imatinib treatment was ongoing, the patient was asymptomatic and there was no radiological or clinical evidence of disease recurrence.

## Discussion

Gastric GIST is a relatively rare entity of nonepithelial, mesenchymal neoplasm with increasing rates of diagnosis due to the widespread use of upper endoscopy and endoscopic ultrasound. Gastric GIST may grow quite large without any symptoms. In the series of De Vogelaere *et al*, GISTs with a tumor size larger than 2 cm accounted for >80% of cases (6). Partial gastrectomy with a gross free surgical margin is accepted worldwide as a treatment method since GIST rarely involves the lymph nodes. The role of laparoscopic, even single-incision laparoscopic surgery, has been established in the past decade in the treatment of gastric GIST (1–4,7,8). However, the feasibility of laparoscopic resection for large gastric GISTs remains largely unknown. The GIST Consensus Conference 2004 (9) recommended limiting laparoscopic resection to tumors smaller than 2 cm due to the increased risk of tumor rupture and spillage. The consensus of Chinese experts is the recommendation of laparoscopic surgery for GIST patients with a tumor size less than 5 cm. Liver metastases are common in advanced or unresectable GIST; however, these metastases may be curable when the primary disease has been eradicated and negative surgical resection margins are attained. Although evidence is lacking, literature reports of colorectal liver metastases have demonstrated that one-stage resection for primary tumor and liver metastases is safe and effective (10).

In our case, surgical resection was not selected as the initial therapy for the following reasons. First, the anatomical location and the association between the giant primary tumor and the contiguous organs was not clear, with some doubt regarding the invasion to the diaphragm and splenic hilum. Extended surgery would be required without any appreciable gain in clinical benefit. Second, as multiple liver metastases were present in the different liver segments, surgery may have led to a marked loss of organ function. Third, genetic testing of biopsy specimens revealed the c-kit exon 11 mutation which indicated a notable response to imatinib therapy (11,12). Guidelines from the National Comprehensive Cancer Network (NCCN) also recommend preoperative imatinib treatment as the primary treatment for metastatic GIST. After 6 months’ therapy, a curative surgery synchronously resecting primary tumor and liver metastases was enabled.

Owing to the cooperation of oncologists and surgeons, our patient received curative treatment with visceral and functional preservation, maintaining a high quality of life.

One-stage laparoscopic resection may be an option for large gastric GISTs with synchronous liver metastases. For advanced or unresectable GIST, preoperative imatinib treatment may be a bridge to curable disease. Minimally invasive approaches may be as safe and effective as open surgery. A greater number of cases and long-term follow-up are necessary to confirm this.
